# MRI utility in the detection of disease activity in clinically stable patients with multiple sclerosis: a retrospective analysis of a community based cohort

**DOI:** 10.1186/s12883-016-0699-8

**Published:** 2016-09-22

**Authors:** Stanley Cohan, Chiayi Chen, Elizabeth Baraban, Tamela Stuchiner, Lois Grote

**Affiliations:** 1 Providence Multiple Sclerosis Center, 9135 SW Barnes Rd Suite 461, Portland, 97225 OR USA; 2 Providence Brain and Spine Institute, 9155 SW Barnes Rd Suite 304, Portland, 97225 OR USA; 3 Providence Brain and Spine Institute, 9155 SW Barnes Rd Suite 731, Portland, 97225 OR USA

**Keywords:** Relapsing multiple sclerosis, MRI, Disease modifying therapy

## Abstract

**Background:**

Since the application of MRI scanning to the diagnosis and treatment of multiple sclerosis, it has been recognized that only a small fraction of lesions seen on MRI scans produce recognizable symptoms or neurological findings. Because new lesions may occur without clinical detection, the recommendation has been made that MRI scanning be performed on a routine scheduled basis, usually yearly, even in patients who are clinically stable.

**Methods:**

A retrospective chart review study was conducted on MS patients who had MRI scans of the central nervous system between 2009 and 2012 at Providence Multiple Sclerosis Center. Inclusion criteria were patients with relapsing MS who had been treated with interferon beta or glatiramer acetate for 6 months or longer. Information on type, indication, and result of MRI and whether a change in disease modifying therapy occurred as a result of the scan was collected.

**Results:**

Of the 436 clinically stable patients who had routine MRI, 16.7 % of subjects had scans revealing new, enlarged or active lesions, yet in only 4.4 % patients was there a change in therapy based upon MRI results. Subjects who had MRI changes were found to be younger (50.15 vs 53.43, *p* = 0.02) but there was no significant difference in other demographic or clinical characteristics when compared with the subjects who did not have MRI changes. Thirty-six percent of patients with MRI changes did not change DMT due to patient request.

**Conclusions:**

This study provides data on the likelihood of detecting MRI-documented disease activity, in patients demonstrating longer term sustained clinical stability while receiving DMTs. These results may materially assist in the decision whether or not to perform yearly MRI scanning of such patients. The potential clinical impact of the results of routine MRI scanning must be weighed against the consideration of considerable expense of frequent MRI scanning, and the yet unknown adverse impact of retained gadolinium in patients repeatedly receiving this contrast agent. The long-term clinical impact of not changing DMTs in patients in whom MRI changes were observed will be addressed in future studies of this cohort.

## Background

For patients with multiple sclerosis (MS), the advent of MRI scanning has resulted in significant improvement in the ability to accurately diagnose the disease [1–7], and has provided important insights into disease activity [3, 8], prognosis [9–13], and response to therapeutic agents [8, 14, 15]. It has also been demonstrated that MRI is a more sensitive indicator of on-going disease activity [2, 3] and burden of disease [2, 3] than symptom reporting or clinical neurological examination. Accordingly, MRI scanning has assumed a major role in routine management and therapeutic decision making in patients with MS. It is the practice of many clinicians to obtain periodic MRI scanning in clinically stable patients, who are being treated with disease modifying therapies (DMT), to detect the presence of clinically silent disease activity. This practice has also been endorsed on the basis of expert opinion [16], but we are unaware of any data that support this practice, particularly in clinically stable patients treated with DMTs for more than 1 to 2 years. It had been our experience, in the community out-patient setting, that the number of MRI scans showing significant change, in clinically stable MS patients receiving DMT, were few in number, leading us to question the value of performing such scans on a routine basis and serving as the rationale for the retrospective data analysis which constitutes this report.

## Methods

In this retrospective study, data from medical records of patients who received their care at the Providence Multiple Sclerosis Center (PMSC) were collected and analyzed. All patients received comprehensive MS care from PMSC neurologists who were experts in the diagnosis and care of patients with MS. Each recorded clinic visit included a complete interval symptom history, and a full neurological examination, with standard clinical information obtained by the examining neurologist. All records were contained in a central electronic medical record system. Chart review and data collection were done by a registered nurse with extensive experience in MS, using a standardized acquisition form and database. Although it was not possible in the community setting to assure that every patient was examined on the same MRI scanner, all patients were studied using a 1.5 or 3.0 tesla MRI scanner, utilizing a standardized MS protocol, with 5 mm contiguous gap-free slices of the brain. All scans were interpreted by neuroradiologists with extensive experience in diagnostic imaging for MS, and also by the MS neurologist caring for the patient. Brain MRI sequences included T1 and T2 weighted axial, FLAIR axial and sagittal, diffusion weighted axial, and gadolinium-enhanced axial views in each patient. Spinal sequences included T1, T2, STIR, FRFSE and T1 gadolinium-enhanced sequences in 4 mm sagittal and 3 mm axial planes. There was at least a 5 min delay following completion of gadolinium infusion before obtaining the T1 weighted enhanced images.

Patients were included in the chart review if they were 18 years or older, had been diagnosed with MS and had been receiving interferon beta (IFN-B) or glatiramer acetate (GA) at PMSC between January 1, 2009 and December 31, 2012. The study was approved by Providence Institutional Review Board. The IRB granted waiver of consent because the study utilized existing records that had been collected for non-research purposes and the data had been de-identified before they were used for the analysis of this study.

**Fig. 1:**
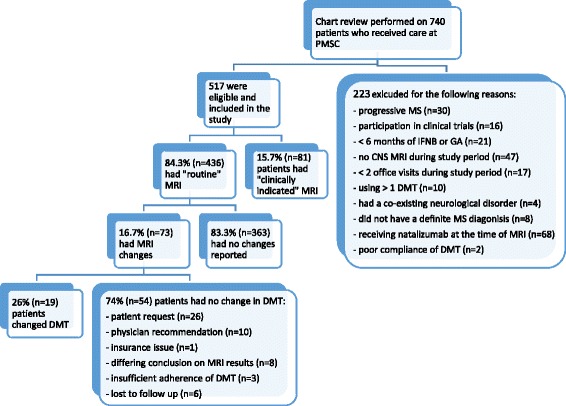
Disposition of the study population

Of the 740 patients eligible for chart review, 223 were excluded from the analysis for the following reasons: progressive forms of MS [16] (*n* = 30), participation in any investigational drug trial (*n* = 16), had not received IFN-B or GA for at least 6 months (*n* = 21), did not have any central nervous system (CNS) MRI performed between 2009 and 2012 (*n* = 47), did not have two or more office visits during the 4 year observation period (*n* = 17), were concurrently receiving more than one MS DMT, excluding those who received intravenous corticosteroids for a relapse, (*n* = 10), had a co-existing neurological disorder (*n* = 4), did not have a definite diagnosis of MS by McDonald criteria (*n* = 8), or had received IFN-B or GA between 2009 and 2012 but were being treated with natalizumab at the time of the MRI of interest (*n* = 68). Another two patients were removed from the study due to poor compliance of their DMT determined by the treating physician. The final sample consisted of 517 patients with relapsing MS, including those with a single clinical event (Clinically Isolated Syndrome or CIS). The disposition of the study cohort is presented in Fig. 1.

All MRI results obtained between January 1, 2009 and December 31, 2012 were reviewed. Scans were categorized into the following: “routine” if they were not performed in response to new or altered symptoms reported by the patient or changes observed in the neurological examination, or “clinically indicated” if they were obtained in response to changes in neurological signs and/or symptoms. Scans were also categorized as brain, cervical or thoracic spinal cord. Additional data obtained included patient demographics, time since diagnosis of MS, duration of IFN-B or GA use, relapse history, date, reason and type of MRI performed, MRI results, DMT change and reason for DMT change following scan results.

Demographic and clinical characteristics of patients were expressed as number, mean and standard deviation or percentage, depending on data type. The number and proportion of “routine” MRI scans that revealed changes or resulted in a switch in DMT were calculated overall and by MRI scan and lesion type. Lesions were characterized as changed if there was reported enlargement of pre-existing lesions, new lesions or gadolinium enhancing lesions. T-tests or non-parametric tests, depending on data distribution, and chi-square tests were used to analyze differences between patients with and without MRI changes on routine scans. P-values less than 0.05 were considered significant. Statistical analyses were performed utilizing SPSS version 22.0.

## Results

The study cohort (*n* = 517) had a mean age of 51.2 (±11.7) years (range 20–83) and was predominantly female (82 %). Mean duration of disease was 12.0 (±7.5) years (range 2–46). At baseline, 94.2 % of patients (*n* = 487) had RRMS and 5.8 % (*n* = 30) had CIS. The distribution of MS therapies were: 77.8 % (*n* = 402) on IFN-B 1a, 20.8 % (*n* = 108) on GA, and 1.4 % (*n* = 7) on IFN-B 1b. The mean duration of therapy on their current DMT was 5.73 (±3.75) years (range 0.6–20). The demographic and clinical characteristics of patients in the study cohort who had routine MRI are shown in Table 1.

**Table 1:** Patient characteristics and demographics

Description	Mean (SD) or %	Number
Age, mean years (SD)	51.2 (11.7)	517
Female, %	82	424
MS Pattern, %		
Relapsing Remitting MS	94.2 %	487
Clinically Isolated Syndrome	5.8 %	30
Duration of Disease, mean years (SD)	12 (7.5)	363
Years of Current DMT Use, mean (SD)	5.73 (3.75)	432
Health Insurance, %		
Private	81.5	422
Medicare	12.4	64
Public	3.1	16
Other	0.2	1
None	2.5	13
Current DMT, %		
IFNB 1a	77.8	402
IFNB 1b	1.4	7
GA	20.8	108

*Abbreviations*: *DMT* disease modifying therapy, *IFNB* interferon beta, *GA* glatiramer acetate

Mean and median observation times included for this study were 2.65 and 3.17 years, respectively. An average of 0.85 MRI was performed per patient per year (on average an MRI every 14.1 months). For the 517 patients included in the study, a total of 1,347 scans were obtained: 64 % brain (*n* = 871), 25 % cervical spine (*n* = 335) and 11 % thoracic-lumbar spine (*n* = 141). Sixty-six percent of the scans (*n* = 886) were ordered in 436 patients who had been clinically stable (“routine”). Of the 436 patients for whom “routine” MRI results were reviewed, changes were observed on 73 patients (16.7 %). Of these, 94.5 % (*n* = 69) had new lesions, 24.7 % (*n* = 18) had enhancing lesions, and 16.4 % (*n* = 12) had enlarged lesions. However, only 26.0 % (*n* = 19) of these patients subsequently changed DMT due to the MRI results, representing only 4.4 % of the 436 patients who had “routine” MRIs. The 54 patients did not change DMT after routine MRI revealed changes for the following reasons: 26 by patient request, 10 by physicians’ recommendation, 1 for an insurance issue, 8 due to differing conclusions of the MRI results between the MS neurologist and the neuroradiologist. In addition, 3 patients not initially excluded, were considered insufficiently adherent in their use of MS medication. Another 6 patients were lost to follow up after their last MRI scan; therefore, we could not confirm DMT change.

There were a total of 54 new and/or enhancing lesions observed in the 62 scans obtained in the 54 patients who did not change DMT. The locations of the 54 lesions are presented in Table 2. Of these lesions, 5 were enhancing, with the largest being a non-enhancing lesion 9 mm in its greatest diameter (a subcortical non-enhancing white matter lesion in a patient electing not to change medication) and all other lesions being 5 mm or less in their greatest diameter. Of the 10 patients in whom physicians elected not to change medication based upon MRI changes, 14 new lesions were detected, 8 of which were 4–5 mm in their greatest diameter and 6 of which were 2–3 mm in diameter. However, none were enhancing, enlarging, or in the spinal cord or brain stem.

**Table 2:** Localization and frequency of lesions in patients with MRI findings but did not change DMT

Location	No. (total *n* = 54)
Frontoparietal subcortical or deep white matter	26
Frontal juxtacortical	1
Deep optic radiations	1
Internal capsule	4
Periventricular	9
Pericallosal	3
Corpus callosum	3
Optic nerve	1
Cerebellum	2
Cervical spinal cord	4

The patients whose routine MRI revealed changes were younger (50.15 vs 53.43 years old, *p* = 0.02) than those with stable MRI. No significant differences were found in disease duration (*p* = 0.42), DMT used (*p* = 0.58), duration of medication use (*p* = 0.53), disease pattern (*p* = 0.17), gender (*p* = 0.93), or type of health insurance (*p* = 0.46) between the two groups (Table 3).

**Table 3:** Comparisons of demographic and clinical characteristics between patients with and without MRI changes on routine scans

	With change (*n* = 73)	Without change (*n* = 363)	*p*
Age, years	50.15	53.43	0.023
Female, %	82.4	82	0.929
MS Pattern, %			0.172
Relapsing Remitting MS	97.3	93.1	
Clinically Isolated Syndrome	2.7	6.9	
Duration of Disease, years	11.39	12.17	0.424
Current DMT, %			0.584
IFNB-1a	81.1	76.3	
IFNB-1b	2.7	1.4	
GA	16.2	22.3	
Yeas of Current DMT Use, mean	5.55	6.06	0.53

*Abbreviations*: *DMT* disease modifying therapy, *IFNB* interferon beta, *GA* glatiramer acetate

## Discussion

Studies have demonstrated that the use of MRI scanning increases the sensitivity and specificity of diagnosis in MS [1–7, 17]. MRI is now an important element in widely utilized MS diagnostic criteria [17], as well as a measure of efficacy in clinical trials [13, 14, 17–27]. Furthermore, use of MRI in patients treated with IFN-B, particularly during the first 1–2 years of initiating medication, may be a predictor of prognosis and response to therapy [7–11]. Although yearly use of MRI scans in patients with relapsing MS is recommended by expert opinion [2, 6], we are unaware of any data that supports the routine yearly use of MRI scanning in clinically stable patients receiving DMTs over the longer term. In our cohort of clinically stable patients, the mean interval between scans was 14.1 months, and the mean duration of their current therapy was 5.73 years. New, enlarged and/or gadolinium-enhancing lesions were detected in 16.7 % of these clinically stable patients. Even if one excludes the small number of cases in which the neurologist did not agree with the official neuroradiologic diagnosis (*n* = 8), or were retrospectively excluded because of insufficient medication compliance (*n* = 3), 14.4 % of the patients demonstrated MRI evidence of disease activity. With exception of patient age, there were no factors identified which were significantly associated with the risk of a subject exhibiting worsening on MRI in the absence of change in symptoms or findings.

The range of available, meaningful medication changes were limited during the time span of this study, and in the 10 cases in which the neurologist chose not to change DMT, the limited number of alternative choices and concerns for patient safety may have influenced this decision. Over the past several years “no evidence of disease activity” (NEDA) has emerged as the sought after standard for therapeutic outcome in clinical trials as well as in clinical practice, and has become a potentially more achievable outcome as a consequence of the increasing number of new, more selectively targeted DMTs that have become available [28]. Although we do not yet know whether evidence of subclinical disease activity in longer term DMT-treated patients is of comparable prognostic significance to that observed in patients who had shorter treatment durations [7–12], it would be prudent to assume that risk of future clinical disease activity and disability worsening is significant and thus prompt recommendation for change in DMT. MRI scanning more often than yearly might reveal MRI lesions missed by yearly intervals. Although a greater scanning frequency might detect short-lived gadolinium enhancing lesions, this probably would have little to no significant impact on the number of new or enlarged lesions seen on T2-weighted images with yearly scanning.

The strengths of our study include the large cohort size, the community base of patients, and elimination of the potential case selection bias inherent in most prospective controlled clinical trials. In addition, our patient cohort had been closely followed, in one treatment center with expert MS neurologists administering standardized neurological examination and MRI scans and complete clinical and treatment history of the subjects available for review.

Shortcomings of this study include its retrospective design and the inability to insure that every subject was studied on the same MRI scanner longitudinally. There was also an imbalance in the percentage of patients receiving IFN-B vs GA. It is important to note that the results of this study cannot be extended to include medications other than IFN-B and GA, and are not relevant to patients not receiving DMT’s, in whom it has been demonstrated that on-going disease activity occurs in the absence of changes in clinically apparent disease activity [3].

Although contiguous MRI sections were without gaps, the 3–5 mm slice thickness may have resulted in missing some small lesions, and lesion size would have been better characterized if more precise volumetric measurement of each lesion could have been obtained with thinner slices. Although this represents a technical weakness of this study that may have led to under-estimation of disease activity in this population, this work does represent the real world MS clinical center setting, in which most MS patients are cared for, and in which most clinical management decisions regarding MS patient care must be made. We do not believe that these shortcomings significantly affect the validity of our observations, and although the long term clinical impact of subclinical MRI activity is not known, the large percentage (14.4–16.7 %) of patients in this study demonstrating subclinical disease activity supports routine scheduled MRI scanning. While our study reflects the experience of one MS Center, the results should prompt other clinicians to employ yearly MRI scanning in their stable patient population.

## Conclusions

This study demonstrated a high likelihood, up to 16.7 %, of detecting disease activity in clinically stable MS patients being treated with IFN-B or GA, in a community-based cohort in which routine MRI scanning was performed on a yearly basis. This level of clinically undetected disease activity supports the practice of at least yearly routine MRI scanning in clinically stable patients, to strengthen efforts to optimize use of DMTs and increase the number of patients achieving NEDA. These recommendations should be weighed against the considerable expense of frequent MRI scanning and the as yet unknown adverse impact of retained gadolinium in patients repeatedly receiving this contrast agent. The long-term clinical impact of not changing DMTs in patients in whom MRI changes were observed will be addressed in future studies of this cohort.

## Abbreviations

CIS: Clinically isolated syndrome
CNS: Central nervous system
DMT: Disease modifying therapies
GA: Glatiramer acetate
IFN-B: Interferon-beta
MS: Multiple sclerosis
NEDA: No evidence of disease activity
PMSC: Providence Multiple Sclerosis Center
